# Gender, income and mental health: The Turkish case

**DOI:** 10.1371/journal.pone.0232344

**Published:** 2020-04-29

**Authors:** Tekin Kose

**Affiliations:** Department of Economics, TED University, Ankara, Turkey; University of Alicante, SPAIN

## Abstract

Gender gaps in health outcomes are frequently observed. Mental health disorders also display gender differences in various countries. This paper explores gender differences in mental health outcomes of individuals in Turkey. It aims to deliver additional evidence on associations between gender, income and mental health status by providing an empirical analysis from a developing country, Turkey. This study employs a nationally representative data set from Turkish Health Survey of 2016. It constructs an index for mental health at individual level by using polychoric principal component analysis. Conditional mixed process models are estimated for quantification of associations between gender, income and mental health measures. Empirical findings indicate that there is endogenous and positive relationship between household income level and mental health status of individuals in Turkey. Turkish females report lower mental health statuses than Turkish males. Furthermore, females are more likely to use mental health services in Turkey. Gender gaps in both mental health status and mental health service use are present in the Turkish case. Results of this study imply that mental health policies should avoid applying one-fit-all approaches.

## Introduction

Prevalences of mental disorders lead to massive economic burden [1, 2, 3]. 4.4% of the world population experienced depression and 3.6% had anxiety disorders during 2015 [4]. According to the Ministry of Health, 18% of Turkish population experience mental illnesses over their adult lifetime [5]. Gender differences in mental health outcomes are robustly observed [6, 7, 8]. For instance, females are more likely to have depressive symptoms and anxiety disorders [9, 4].

Gender differences in health outcomes may result from biological, psychological, epidemiological, socioeconomic factors and interactions of these conditions. Gender of an individual is associated with her/his socioeconomic conditions, access to various resources, options and treatments in social and economic life. Researchers observe that gender-related social conditions are significantly correlated with gender differences in mental health status [10]. Seedat et al. [9] reveal that gender gaps in mental disorders are consistently observed in various countries from different regions of the world. Earlier studies consistently reveal that women tend to have higher frequencies of mental health disorders [11, 12, 13, 14]. However, gender differences shrink when men and women have more equal roles in the society [9].

Prior studies indicate that demographic conditions of individuals are associated with their psychological well-being. Emergence and prevalence of different mental health disorders vary across age groups of population in many countries [15, 16]. Improvements in education and learning are positively associated with better health outcomes [17]. Educated individuals are more likely to obtain information and seek treatments [18, 19]. Moreover, lower education levels are directly correlated with mental health disorders [20, 21]. Some researchers find that marriage is positively correlated with mental health status [22, 23, 24]. Individual level economic conditions such as employment status and income level are also associated with health outcomes of individuals. Unemployed individuals are more likely to experience mental health problems such as psychological distress and depression [25, 26, 27, 28]. Paul and Moser [29] suggest that unemployed men are more likely to be distressed than unemployed women. Prior research also reveals that mental health outcomes of individuals are correlated with body mass index (BMI), especially for women [30, 31, 32].

There exists a robust association between income and various health outcomes [33, 34, 35, 36, 37, 38]. Researchers identify two-way causality between income and health measures [37, 38, 39, 40]. Bloom and Canning [39] argue that healthy people tend to be more productive; more likely to invest in human capital, hence they are more educated. Additionally, since they are more likely to live longer they tend to invest in physical capital. Thus, healthy people are to generate higher levels of income. On the other hand, higher income lead individuals to access health related resources and services [37, 38]. Finally, another branch of literature reveals that income inequality adversely related with mental health outcomes [40, 41, 42].

Access and use of health care resources are associated with health status of individuals. Existing literature indicates significant differences among men and women in terms of seeking and utilization of mental health care services. Women are more likely to have professional help-seeking attitudes [43, 44, 45]. Moreover, access to social support is a predictor for mental health status of individuals [46, 47, 48]. For instance, Cotten [24] suggests that presence of social support is negatively associated with likelihood of experiencing depressive symptoms. Finally, health related behaviors of individuals such as smoking [49, 50, 51, 52], alcohol use [53, 54], dietary habits [55, 56, 57] and physical activity [55, 57, 58, 59] are associated with mental health outcomes.

This study extends the existing literature by analysing gender differences in mental health status in a developing country, Turkey. It explores associations of demographics, socioeconomic conditions, social factors and health-related behaviors with mental health statuses of Turkish individuals by specifically focusing on gender. In order to quantify sociodemographic and social determinants of mental health, this study utilizes a nationally representative data set from Health Survey of Turkey, which is conducted by Turkish Statistical Institute (TSI) [60].

In order to measure mental health status of individuals, this study employs polychoric principal component analysis (PPCA) and constructs an index for mental health. Mental health status index is based on various measures from Patient Health Questionnaire (PHQ-9) based indicators of mental health. This study utilizes simultaneous equation modelling. Covariates of mental health status index, mental health service use, body mass index, being married and household income level are estimated by conditional mixed-process models (CMP).

Empirical results confirm the use of simultaneous equation framework and indicate that there are gender differences in mental health outcomes in Turkey. Females have lower mental health status than males. Age, smoking frequency and alcohol use frequency are negatively associated with mental health status. Education level, social support, healthy diets and physical activity are positively related with mental health in Turkey. Being employed is positively associated with mental health level of Turkish males only.

Females are more likely to employ mental health services compared to males in Turkey. Age level, smoking frequency and alcohol use frequency display positively significant correlations with likelihood of mental health service use for Turkish females. On the other hand, age level, being employed and social support level are negatively related with probability of mental health service use by Turkish males. Thus, there are both similarities and differences in risk factors of mental health status and mental health service use across sub-samples of gender in Turkey. These findings imply that gender-focused health intervention policies should be designed for reduction of disparities in health.

## Methods

This study employs data from 2016 wave of Turkish Health Survey (THS). This is a nationally representative household survey conducted by TSI. This survey track information on various health related measures and activities of Turkish individuals such as health status, insurance status, use of health care services and lifestyle. The survey utilizes stratified sampling method and includes 9,740 households from different regions of Turkey. The current study considers individuals who are at the age of 15 or older and the operating sample includes 17,242 respondents. Ethical approval and consent agreement for the current study are not required since the data is collected and provided by TSI in line with regulations determined by Statistics Law of Turkey. The author(s) are granted access for an anonymized version of the data set for research purposes.

In order to quantify mental health status at individual level, this study constructs an index by polychoric principal component analysis (PPCA). This index considers categorical measures from PHQ-9 based questions of THS. The survey questions read the following: “Over the last 2 weeks, how often have you been bothered by X [Insert one of the eight categories given below]?” Eight questions are included in the mental health index: 1) “Having little interest or pleasure in doing things”; 2) “Feeling down, depressed”; 3) “Trouble falling or staying asleep, or sleeping too much”; 4) “Feeling tired or having little energy”; 5) “Poor appetite or overeating”; 6) “Feeling bad about yourself or that you are a failure or have let yourself or your family down”; 7) “Trouble concentrating on things, such as reading the things, such as reading the newspaper or watching television”; 8) “Moving or speaking so slowly that other people could have noticed. Or the opposite-being so fidgety or restless that you have been moving around a lot more than usual”. The respondents are asked to choose one of following options: 1-Not at all; 2-Several days; 3-More than half the days; 4-Nearly every day. For PPCA, these measures are rescaled such that 1 represents the highest frequency of mental health indicators and 4 represents the lowest frequency. THS does not ask the 9th question of PHQ-9, which is about “hurting oneself” and “thoughts on suicide”. Thus, this study cannot use standart depression severity measures based on PHQ-9 due to data limitation. By using PPCA, this study combines eight categorical indicators of mental health and construct a continuous index for mental health status of individuals. The mental health status index continuously ranges from 1 to 4, 4 representing the best mental health status. PPCA results indicate single factor solution since there is only one eigenvalue (5.51) which is greater than one. Standard Cronbach’s alpha of index items reads 0.89. Factor loadings of eight components of index from PPCA are ranging from 0.77 to 0.89, all of which exceed the thresholds provided by the literature [61].

Empirical estimation of this study follows the conditional mixed-process (CMP) framework. CMP models allow researchers to estimate multiple equation systems which may include different dependent variables and various independent variables [62]. In this study, mental health status, utilization of mental health care services, body mass index (BMI), marital status and household income level are employed as dependent variables of the simultaneous equation system. Mental health service use is a binary variable which is equal to one if the individual visited a psychologist or a mental therapist or a psychiatrist in the last year. A continuous measure of BMI for the respondent (kg/m^2^) is considered. Marital status is quantified by a binary variable which reads one if the respondent is married. Monthly household income of respondents are categorically measured with five levels represented in Turkish Liras. Income level categories read the following: 1 = 0–1264 TL; 2 = 1265–1814 TL; 3 = 1815–2540 TL; 4 = 2541–3721 TL; 5 = 3722+ TL. Empirical models also include control variables for other behavioral and socioeconomic factors such as age, employment status, presence of social support, alcohol use, smoking behavior, dietary and physical activity habits. Descriptions of all variables included in the study are presented by Table 1. Frequency distributions of variables across gender are provided by Table 2. Summary statistics for variables of interest are given Table 3.

**Table 1:** Description of variables.

*Variable*	*Description*
Little Interest	Measures frequency of being bothered by little interest or pleasure in doing things over last 2 weeks for the individual. 1-Not at all; 2-Several days; 3-More than half the days; 4-Nearly every day.
Feeling Depressed	Measures frequency of being bothered by feeling down, depressed over last 2 weeks for the individual. 1-Not at all; 2-Several days; 3-More than half the days; 4-Nearly every day.
Sleeping Problem	Measures frequency of being bothered by trouble falling or staying asleep, or sleeping too much over last 2 weeks for the individual. 1-Not at all; 2-Several days; 3-More than half the days; 4-Nearly every day.
Feeling Tired	Measures frequency of being bothered by feeling tried or having little energy over last 2 weeks for the individual. 1-Not at all; 2-Several days; 3-More than half the days; 4-Nearly every day.
Eating Problem	Measures frequency of being bothered by poor appetite or overeating over last 2 weeks for the individual. 1-Not at all; 2-Several days; 3-More than half the days; 4-Nearly every day.
Feeling Invaluable	Measures frequency of being bothered by feeling bad about himself/herself or that s/he is a failure or have let himself/herself or his/her family down over last 2 weeks for the individual. 1-Not at all; 2-Several days; 3-More than half the days; 4-Nearly every day.
Concentration Problem	Measures frequency of being bothered by trouble concentrating on things, such as reading the things, such as reading the newspaper or watching television over last 2 weeks for the individual. 1-Not at all; 2-Several days; 3-More than half the days; 4-Nearly every day.
Having Discomfort	Measures frequency of being bothered by by moving or speaking so slowly that other people could have noticed or the opposite-being so fidgety or restless that s/he has been moving around a lot more than usual over last 2 weeks for the individual. 1-Not at all; 2-Several days; 3-More than half the days; 4-Nearly every day.
Mental Health Index	This index is constructed by Polychoric Principal Component Analysis framework. 8 indicators of mental health (Little Interest, Feeling Depressed, Sleeping Problem, Feeling Tired, Eating Problem, Feeling Invaluable, Concentration Problem, Having Discomfort) are rescaled such that higher values of variables represent better mental health. Rescaled measures are utilized for the index. The mental health status index continuously ranges from 1 (the worst mental health level) to 4 (the best mental health level).
Female	1 = Female; 0 = Male.
Age Level	Reported age level group of the respondent. Age levels are: 1 = 15–24; 2 = 25–34; 3 = 35–44; 4 = 45–54; 5 = 55–64; 6 = 65–74; 7 = 75+
Education Level	Reported highest educational attainment of the respondent. Education groups are: 0 = Illiterate; 1 = No Official Diploma; 2 = Primary School; 3 = Secondary School; 4 = High School; 5 = Associate Degree; 6 = Bachelor’s Degree; 7 = Graduate Degree
Employed	Working status of the respondent. 1 = Employed; 0 = Otherwise
Married	Measures marital status of the respondent. 1 = Married; 0 = Otherwise.
Body Mass Index (BMI)	Continuous measure of BMI for the respondent (kg/m^2^).
Mental Health Service Use	1 = The respondent visited a psychologist or a mental therapist or a psychiatrist in last 12 months. 0 = Otherwise.
Reliable Friends	Categorically measured number of reliable, close friends the respondent can count on if s/he has serious personal problems. 1 = None; 2 = 1 or 2; 3 = 3 to 5; 4 = 6 or more.
Interest of Others	Measures degree of the concern shown by others in what the respondent is doing. 1 = No Concern and Interest; 2 = Little Concern and Interest; 3 = Uncertain; 4 = Some Concern and Interest; 5 = A Lot of Concern and Interest
Neighbor Help	Measures easiness of getting help from the respondent’s neighbors if needed. 1 = Very Difficult; 2 = Difficult; 3 = Possible; 4 = Easy; 5 = Very Easy
Smoking Frequency	Measures smoking frequency of the respondent.0 = Non-Smoker; 1 = Ex-Smoker; 2 = Occasional Smoker; 3 = Daily Smoker
Alcohol Use Frequency	Measures alcohol use frequency of the respondent.0 = Non-Drinker; 1 = Ex/Rare Drinker; 2 = Occasional Drinker; 3 = Drinker; 4 = Frequent Drinker
Walking Days	The number of days in which respondent spends more than 10 minutes on walking during a typical week.
Vegetable Consumption Frequency	Measures frequency of eating vegetables. 0 = Never; 1 = Less than once a week; 2 = 1 to 3 times a week; 3 = 4 to 6 times a week; 4 = Once or more a day
Fruit Consumption Frequency	Measures frequency of eating fruits. 0 = Never; 1 = Less than once a week; 2 = 1 to 3 times a week; 3 = 4 to 6 times a week; 4 = Once or more a day
Household Income	Reported monthly household income level category of the respondent (in Turkish Liras). 1 = 0–1264 TL; 2 = 1265–1814 TL; 3 = 1815–2540 TL; 4 = 2541–3721 TL; 5 = 3722+ TL.
Household Size	Measures the number of individuals in the household in which the respondent lives in.

Source: TSI (2016).

**Table 2:** Frequency distributions of variables across gender.

	Males		Females	
Variables	N	% or *Mean (s*.*d*.*)*	N	% or *Mean (s*.*d*.*)*
Little Interest: Not at all	5,432	70.84	5,612	58.62
Several days	1,920	25.04	3,343	34.92
More than half the days	115	1.50	234	2.44
Nearly every day	201	2.62	385	4.02
Feeling Depressed: Not at all	4,917	64.12	5,035	52.59
Several days	2,337	30.48	3,758	39.25
More than half the days	162	2.11	320	3.34
Nearly every day	252	3.29	461	4.82
Sleeping Problem: Not at all	5,549	72.37	5,658	59.10
Several days	1,611	21.01	2,895	30.24
More than half the days	165	2.15	363	3.79
Nearly every day	343	4.47	658	6.87
Feeling Tired: Not at all	4,619	60.24	4,338	45.31
Several days	2,513	32.77	4,142	43.26
More than half the days	208	2.71	421	4.40
Nearly every day	328	4.28	673	7.03
Eating Problem: Not at all	6,192	80.75	6,785	70.87
Several days	1,204	15.70	2,238	23.38
More than half the days	89	1.16	202	2.11
Nearly every day	183	2.39	349	3.65
Feeling Invaluable: Not at all	6,484	84.56	7,005	73.17
Several days	1,000	13.04	2,151	22.47
More than half the days	68	0.89	162	1.69
Nearly every day	116	1.51	256	2.67
Concentration Problem: Not at all	6,562	85.58	7,406	77.36
Several days	927	12.09	1,809	18.89
More than half the days	79	1.03	155	1.62
Nearly every day	100	1.30	204	2.13
Having Discomfort: Not at all	6,899	89.97	8,064	84.23
Several days	638	8.32	1,264	13.20
More than half the days	45	0.59	100	1.04
Nearly every day	86	1.12	146	1.52
Mental Health Index	7,668	*3*.*69 (0*.*46)*	9,574	*3*.*55 (0*.*54)*
Mental Health Service Use	7,668	3.56	9,574	6.26
Age Level: 15–24	1,344	17.53	1,561	16.30
25–34	1,269	16.55	1,737	18.14
35–44	1,508	19.67	1,936	20.22
45–54	1,373	17.91	1,634	17.07
55–64	1,055	13.76	1,313	13.71
65–74	702	9.15	843	8.81
75+	417	5.44	550	5.74
Education Level: Illiterate	211	2.75	1,483	15.49
No Official Diploma	274	3.57	680	7.10
Primary School	2,623	34.21	3,325	34.73
Secondary School	1,559	20.33	1,417	14.80
High School	1,656	21.60	1,450	15.15
Associate Degree	416	5.43	395	4.13
Bachelor’s Degree	798	10.41	726	7.58
Graduate Degree	131	1.71	98	1.02
Employed	4,399	57.37	2,058	21.50
Married	5,417	70.64	6,495	67.84
Household Income: 0–1264 TL	1,430	18.65	2,241	23.41
1265–1814 TL	2,115	27.58	2,592	27.07
1815–2540 TL	1,426	18.60	1,726	18.03
2541–3721 TL	1,381	18.01	1,563	16.33
3722 + TL	1,316	17.16	1,452	15.17
Household Size	7,668	*2*.*81 (1*.*29)*	9,574	*2*.*77 (1*.*34)*
Body Mass Index (BMI)	7,668	*26*.*07 (4*.*35)*	9,574	*26*.*67 (5*.*69)*
Interest of Others: No Concern and Interest	277	3.61	338	3.53
Little Concern and Interest	655	8.54	809	8.45
Uncertain	1,827	23.83	2,122	22.16
Some Concern and Interest	3,950	51.51	5,084	53.10
A Lot of Concern and Interest	959	12.51	1,221	12.75
Neighbour Help: Very Difficult	290	3.78	318	3.32
Difficult	693	9.04	753	7.87
Possible	1,129	14.72	1,253	13.09
Easy	4,337	56.56	5,559	58.06
Very Easy	1,219	15.90	1,691	17.66
Smoking Frequency: Non-Smoker	2,635	34.36	7,086	74.01
Ex-Smoker	1,735	22.63	711	7.43
Occasional Smoker	326	4.25	398	4.16
Daily Smoker	2,972	38.76	1,379	14.40
Alcohol Use Frequency: Non-Drinker	4,509	58.80	8,506	88.84
Ex/Rare Drinker	1,648	21.49	525	5.48
Occasional Drinker	1,073	13.99	466	4.87
Drinker	345	4.50	73	0.76
Frequent Drinker	93	1.21	4	0.04
Walking Days	7,668	*4*.*93 (2*.*73)*	9,574	*3*.*84 (2*.*85)*
Vegetable Consumption: Never	58	0.76	51	0.53
Less than once a week	237	3.09	210	2.19
1 to 3 times a week	1,309	17.07	1,377	14.38
4 to 6 times a week	1,470	19.17	1,869	19.52
Once or more a day	4,594	59.91	6,067	63.37
Fruit Consumption: Never	115	1.50	159	1.66
Less than once a week	449	5.86	559	5.84
1 to 3 times a week	1,828	23.84	2,109	22.03
4 to 6 times a week	1,311	17.10	1,557	16.26
Once or more a day	3,965	51.71	5,190	54.21

Source: TSI (2016).

**Table 3:** Summary statistics.

*Variable*	*N*	*Mean*	*Standard Deviation*	*Min*	*Max*
Mental Health Index	17,242	3.612	0.510	1	4
Female	17,242	0.555	0.497	0	1
Age Level	17,242	3.431	1.758	1	7
Education Level	17,242	2.842	1.674	0	7
Employed	17,242	0.374	0.484	0	1
Married	17,242	0.691	0.462	0	1
Household Income	17,242	2.793	1.377	1	5
Household Size	17,242	2.788	1.318	1	13
Body Mass Index (BMI)	17,242	26.40	5.145	12.487	66.406
Mental Health Service Use	17,242	0.051	0.219	0	1
Reliable Friends	17,242	2.701	0.846	1	4
Interest of Others	17,242	3.621	0.934	1	5
Neighbor Help	17,242	3.757	0.949	1	5
Smoking Frequency	17,242	0.983	1.271	0	3
Alcohol Use Frequency	17,242	0.399	0.795	0	4
Walking Days	17,242	4.326	2.848	0	7
Vegetable Consumption Frequency	17,242	3.392	0.884	0	4
Fruit Consumption Frequency	17,242	3.138	1.057	0	4

Source: TSI (2016).

The estimation framework of this study follows a simultaneous equation system in which mental health status, mental health service use, BMI, marital status and household income levels are considered as dependent variables. Theoretical equation system is presented below.

$$MH_{i}=X_{i}\theta +u_{i}$$ (1)

$$MHSU_{i}=W_{i}\beta +\omega_{i}$$ (2)

$$BMI_{i}=H_{i}\alpha +\varepsilon_{i}$$ (3)

$$M_{i}=K_{i}\delta +e_{i}$$ (4)

$$IL_{i}=Z_{i}\phi +v_{i}$$ (5)

MH_i_ denotes mental health index, MHSU_i_ denotes mental health service use status, BMI_i_ represents body mass index, M_i_ denotes marital status and IL_i_ denotes household income level for individual i. Error terms are normally distributed with pair-wise correlation coefficients ρ’s. X_i_, W_i_, H_i_, K_i_ and Z_i_ are vector of covariates for each dependent variable, respectively. θ = (θ_1_,θ_2_,…..,θ_k_), β = (β_1_,β_2_,…..,β_j_), α = (α_1_,α_2_,…..,α_s_), δ = (δ_1_,δ_2_,…..,δ_r_) and ϕ = (ϕ_1_,ϕ_2_,…..,ϕ_m_) denote vectors of parameters for corresponding equations. Since mental health status index and BMI are continuous variables, these equations are estimated by ordinary least squares (OLS) method. Equations for mental health service use and marital status are estimated by binary probit framework. The last equation for household income is predicted by ordered probit method since household income level is an ordered variable. This equation system is estimated by STATA 15 by utilizing CMP procedures provided by Roodman [62]. CMP jointly estimates the simultaneous equation system and correlation coefficients between error terms by conditional maximum likelihood technique. The simultaneous equation systems are estimated for the full sample and sub-groups of females and males in order to analyze gender differences in mental health.

## Results

First, the average mental health index score of females (3.546 out of 4) is lower than the average mental health index of males (3.694 out of 4) in Turkey. A simple ordinary least squares regression estimation reveals that this raw gender gap in mental health status (-0.148) is statistically significant at 1% level with t-value of -19.49. These results indicate that the raw gender gap corresponds to 3.7% ((-0.148)*100/4) in Turkey. This finding is highly similar with earlier findings from other countries such as and Ireland and Serbia where researchers observe gender gaps in mental health scores of individuals ranging from 2.8% to 4.6%, respectively [63, 64].

Empirical results for CMP models are presented by Table 4 for full sample, Table 5 for female sample and Table 6 for male sample. All regression models are estimated with robust standard errors. Confirming earlier literature [37, 38, 39, 40], estimation results of CMP model reveal that there is endogeneity between household income level and mental status index of individuals in Turkey. The conditional mixed process estimation results exhibit that correlations between error terms of mental health index and household income equations (atanhrhos) are positively significant for all samples. This finding implies that there exist unobserved factors which are positively related with both mental health index and household income level of individuals. Similarly, atanrhos between mental index and being married are significant and positive for all samples. Namely, there are unobserved correlates which are positively associated with both being married and having better mental health level. This finding is in line with previous research which reports positive associations with mental health outcomes and being married [23, 24]. Additionally, correlations of error terms for equations of mental health index and mental health service use are negatively significant for all samples. There are unobserved variables which are negatively connected with both mental health index and likelihood of mental health service use of individuals. Finally, body mass index and mental health status display endogeneity for only full sample and sample of females with negatively significant atanrhos. This observation is line with earlier findings [30, 31], which report gender-specific associations between BMI measures and mental health status of individuals. Overall, empirical results of this study indicate that single equation models would fail to quantify the relationships between mental health status, body mass index, marital status and househol income.

**Table 4:** CMP estimation results for mental health and income: Full sample.

Variables	*Mental Health*	*Mental Health Service Use*	*Body Mass Index*	*Married*	*Household Income*
Female	-0.146***	0.335***	0.408***	0.0532**	
	(0.00877)	(0.0401)	(0.0824)	(0.0222)	
Age Level	-0.0300***	0.00194	0.885***	0.201***	0.0911***
	(0.00243)	(0.0102)	(0.0233)	(0.00699)	(0.00547)
Education Level	0.0279***	0.0225**	-0.304***	-0.0254***	0.361***
	(0.00253)	(0.0115)	(0.0246)	(0.00702)	(0.00617)
Employed	0.0429***	-0.161***	0.862***	0.565***	0.228***
	(0.00791)	(0.0405)	(0.0807)	(0.0230)	(0.0174)
Household Size					0.142***
					(0.00753)
Reliable Friends	0.0496***	-0.0624***			
	(0.00449)	(0.0206)			
Interest of Others	0.0227***	0.0115			
	(0.00471)	(0.0191)			
Neighbor Help	0.0477***	-0.0298*			
	(0.00472)	(0.0178)			
Smoking Frequency	-0.0360***	0.104***	-0.126***		
	(0.00333)	(0.0141)	(0.0314)		
Alcohol Use Frequency	-0.0164***	0.0494**	-0.0120		
	(0.00483)	(0.0220)	(0.0466)		
Walking Days	0.0165***		-0.112***		
	(0.00140)		(0.0136)		
Vegetable Consumption Frequency	0.0178***		0.0474		
	(0.00516)		(0.0470)		
Fruit Consumption Frequency	0.0471***		0.134***		
	(0.00442)		(0.0394)		
Constant	3.067***	-1.767***	23.71***	-0.330***	
	(0.0309)	(0.108)	(0.204)	(0.0413)	
R^2^/Pseudo R^2^	0.122	0.028	0.138	0.082	0.085
Atanhrho (with mental health index)		-0.277***	-0.018**	0.071***	0.073***
		(0.012)	(0.009)	(0.011)	(0.009)
Wald χ^2^	10,155.6***				
Number of Observations	17,242				

Source: TSI (2016).

*** p<0.01

** p<0.05

* p<0.1. Robust standard errors are in parentheses.

**Table 5:** CMP estimation results for mental health and income: Female sample.

Variables	*Mental Health*	*Mental Health Service Use*	*Body Mass Index*	*Married*	*Household Income*
Age Level	-0.0405***	0.0228*	1.010***	0.0325***	0.101***
	(0.00353)	(0.0128)	(0.0353)	(0.00912)	(0.00781)
Education Level	0.0343***	0.00410	-0.456***	-0.0651***	0.355***
	(0.00374)	(0.0147)	(0.0365)	(0.00885)	(0.00837)
Employed	0.0173	-0.0497	0.0493	0.237***	0.296***
	(0.0121)	(0.0529)	(0.127)	(0.0334)	(0.0280)
Household Size					0.166***
					(0.0102)
Reliable Friends	0.0584***	-0.00195			
	(0.00656)	(0.0261)			
Interest of Others	0.0283***	-0.00366			
	(0.00667)	(0.0241)			
Neighbor Help	0.0472***	-0.0179			
	(0.00675)	(0.0225)			
Smoking Frequency	-0.0552***	0.134***	0.0237		
	(0.00553)	(0.0181)	(0.0503)		
Alcohol Use Frequency	-0.0435***	0.0735**	-0.380***		
	(0.0109)	(0.0374)	(0.0973)		
Walking Days	0.0145***		-0.0939***		
	(0.00191)		(0.0192)		
Vegetable Consumption Frequency	0.0201**		0.0622		
	(0.00790)		(0.0718)		
Fruit Consumption Frequency	0.0557***		0.159***		
	(0.00639)		(0.0567)		
Constant	2.896***	-1.647***	24.05***	0.471***	
	(0.0428)	(0.127)	(0.277)	(0.0467)	
R^2^/Pseudo R^2^	0.123	0.019	0.179	0.012	0.087
Atanhrho (with mental health index)		-0.280***	-0.026**	0.056***	0.068***
		(0.016)	(0.011)	(0.013)	(0.012)
Wald χ^2^	5731.87***				
Number of Observations	9,574				

Source: TSI (2016).

*** p<0.01

** p<0.05

* p<0.1. Robust standard errors are in parentheses.

**Table 6:** CMP estimation results for mental health and income: Male sample.

Variables	*Mental Health*	*Mental Health Service Use*	*Body Mass Index*	*Married*	*Household Income*
Age Level	-0.0157***	-0.0319*	0.749***	0.489***	0.0986***
	(0.00334)	(0.0169)	(0.0296)	(0.0138)	(0.00814)
Education Level	0.0245***	0.0492**	0.0442	-0.0108	0.401***
	(0.00364)	(0.0195)	(0.0322)	(0.0126)	(0.00973)
Employed	0.0719***	-0.275***	1.504***	1.145***	0.322***
	(0.0106)	(0.0599)	(0.100)	(0.0360)	(0.0264)
Household Size					0.126***
					(0.0115)
Reliable Friends	0.0383***	-0.166***			
	(0.00598)	(0.0334)			
Interest of Others	0.0175***	0.0279			
	(0.00651)	(0.0315)			
Neighbor Help	0.0466***	-0.0470			
	(0.00650)	(0.0294)			
Smoking Frequency	-0.0200***	0.0729***	-0.194***		
	(0.00404)	(0.0222)	(0.0380)		
Alcohol Use Frequency	-0.0123**	0.0475*	0.193***		
	(0.00526)	(0.0278)	(0.0511)		
Walking Days	0.0188***		-0.0833***		
	(0.00209)		(0.0184)		
Vegetable Consumption Frequency	0.0158**		0.0763		
	(0.00636)		(0.0570)		
Fruit Consumption Frequency	0.0356***		0.123**		
	(0.00579)		(0.0508)		
Constant	3.068***	-1.376***	22.42***	-1.637***	
	(0.0430)	(0.172)	(0.268)	(0.0697)	
R^2^/Pseudo R^2^	0.088	0.038	0.111	0.316	0.089
Atanhrho (with mental health index)		-0.270***	0.004	0.051***	0.087***
		(0.019)	(0.013)	(0.013)	(0.013)
Wald χ^2^	5109.19***				
Number of Observations	7,668				

Source: TSI (2016).

*** p<0.01

** p<0.05

* p<0.1. Robust standard errors are in parentheses.

Results from full sample estimation, presented in Table 4, reveal that there is a gender gap in mental health status of individuals in Turkey. Turkish females have significantly lower mental health index than Turkish males. In line with earlier findings [12, 13], females are to experience higher levels of mental health issues than males in Turkey. According to Tables 4–6, age level is negatively correlated with mental health status of Turkish individuals in all samples. Older individuals are more likely to have lower mental health index in Turkey. Tables 5 and 6 reveal that education level is positively associated with mental health index for both females and males in Turkey. Similar to related literature [20, 21], this study reports that education and mental health outcomes are directly related. Employment status displays different associations with mental health index across sub-groups of gender in Turkey. According to Table 6, being employed is positively related with mental health index of Turkish males. However, Table 5 implies that employment status of Turkish women does not reveal significant correlations with their mental health status. These findings are consistent with results provided by Paul and Moser [29] who indicate that unemployment puts more pressures on men.

According to Tables 5 and 6, findings on associations of mental health outcome and social support conditions are consistent with earlier research [24, 46, 48]. Having more close and reliable friends is positively correlated with better mental health status for both males and females in Turkey. Individuals who are able to easily get help from their neighbors report better mental health measures. Receiving higher level of interest from other people is positively related with higher level of mental health index for residents of Turkey.

Empirical findings, reported in Tables 4–6, indicate that health behaviors are significantly associated with mental health outcomes of Turkish individuals. Individuals with higher frequency of smoking are more likely to have lower levels of mental health status. Unlike previous studies [53, 65], the results of this study indicate a significant association between alcohol use and mental health of females. Higher alcohol use frequency is negatively correlated with mental health levels of both females and males in Turkey. Findings on the relationship of mental health with dietary habits and physical activity are in line with earlier research [55, 57]. Number of walking days in a week, frequency of consuming vegetables and fruit consumption frequency display positive correlations with mental health statuses of females and males in Turkey.

According to Table 4, females are more likely to use mental health services in Turkey. This finding is consistent with earlier literature which reports gender gap in mental health service use [43, 66, 67]. Being employed, having reliable friends and obtaining neighbor help are negatively related with probability of health service use by Turkish individuals. Education level, smoking frequency and alcohol use frequency are positively associated with likelihood of using mental health services in Turkey for the full sample. These findings vary across sub-samples with respect to gender. For instance, education level, employment and social support do not significantly associate with mental health service use for Turkish females. According to Table 5, age level, smoking frequency and alcohol use frequency are positively correlated with mental health service use for Turkish females. Table 6 reveals that age level, being employed, having reliable friends are negative correlates for mental health service use for Turkish males. However, education level, smoking frequency and alcohol use frequency are positively related with likelihood of using mental health services for males in Turkey.

Empirical findings, given in Table 4, imply that Turkish females report significantly higher BMI levels than Turkish males. Tables 4–6 imply that age and fruit consumption levels of Turkish individuals are positively associated with BMI levels in all samples. Physical activity level displays negative relationships with BMI measures of Turkish individuals in all samples. According to Tables 5 and 6, education level is negatively correlated with BMI for only Turkish females. Tables 5 and 6 indicate that being employed is positively associated with BMI for only Turkish males. Smoking frequency is negatively related with BMI for only males in Turkey. Finally, alcohol use frequency and BMI measures are negatively associated for Turkish females whereas they display positive relationship for Turkish males.

Empirical results suggest that probability of being married is positively associated with age level and being employed for both Turkish males and females. However, Tables 5 and 6 reveal that education level is negatively related with likelihood of being married for only Turkish females. Finally, Tables 4–6 exhibit that household income level is directly associated with household size. Older individuals are more likely to live with higher household income. Education level and being employed are positively correlated with household income level. Findings on correlates of household income level are robust and do not vary across sub-samples of gender.

## Conclusion

Health disparities put significant economic burden for the society. Earlier researches from various countries reveal significant gender differences in health outcomes. This study extends the current literature on health disparities in mental health outcomes by providing evidence from Turkey. It uses most recent version of a nationally representative health survey and constructs a mental health status index at individual level, which is based on various indicators of mental health such as experiencing eating and sleeping disorders; feeling invaluable, hopeless, restless, tired, depressed, unsatisfied; and losing concentration in daily activities.

This study utilizes simultaneous equation framework to estimate associations between mental health status, mental health service use, body mass index, being married and household income level. Conditional mixed-process models are estimated for quantification of associations between dependent variables and their correlates such as demographics, social support and health behaviour. Empirical results reveal that there is a significant gender gap in mental health status in Turkey. Females have lower mental health index than males. Gender differences also extend to use of mental health services in Turkey. Turkish females are more likely to utilize mental health services compared to Turkish males. These findings are in line with earlier results reported from various countries. There are positive correlations of mental health with household income level and being married. Mental health status and mental health service use are negatively associated. For females, body mass index and mental health status display negative correlations.

There are both similarities and differences in risk factors of mental health outcomes for males and females in Turkey. Age level, frequency of alcohol use and frequency of smoking are negatively associated with mental health status of individuals regardless of gender. Education level, level of social support, being physically active and having healthy diets are positively correlated with mental health index of Turkish individuals. Employed males are more likely to have better mental health status than males who are not working. Age level, smoking frequency and alcohol use frequency have positively significant associations with use of mental health services for Turkish females. For Turkish males, age level, being employed and social support display negatively significant relations with probability of using mental health services.

Although this study enhances knowledge on associations of gender, income and mental health outcomes, it is important to point out its limitations. First, this study employs survey data and uses self-reported measures which are subjective and sensitive to reporting biases. Second, the constructed mental health index is useful in aggregation of information at the expense of losing details on specific mental health measures. Third, this study only covers a cross-sectional data analysis. Finally, the current study employs simultaneous equation framework and empirical analysis only provides correlations among variables of interest. Hence, findings of this study should be interpreted accordingly. Future studies should focus of identification causal pathways between mental health status and its correlates with cross-national data. Analysis of time dimension for mental health status with longitudinal data would provide significant insights.

Findings of this study have crucial implications for mental health policy interventions. Public health policies should consider the fact that males and females may have different correlates of mental health measures. For instance, unlike males, mental health status of Turkish females is significantly related with body mass index. Employment status is only a correlate of mental health status for males. Hence, addressing gender disparities in health for developing regions of the world would require gender-specific policy designs and applications. Policy makers should avoid one-fit-all health interventions. Overall, this study concludes that gender differences in health outcomes are significantly present and should be immediately addressed by effective policies which specifically targets sub-groups of the society.
